# The efficacy and safety of remimazolam besylate, ciprofol, and propofol during hysteroscopy

**DOI:** 10.3389/fphar.2024.1427755

**Published:** 2025-01-08

**Authors:** Weifeng Shan, Shuying Gao, Mengting Ai, Haiyan Lan, Gongchen Duan, Xiaoli Dong, Qiaomin Xu, Yini Wu, Jimin Wu

**Affiliations:** Department of Anesthesiology, Lishui People’s Hospital, Wenzhou Medical University Lishui Hospital, Lishui, China

**Keywords:** remimazolam, ciprofol, propofol, hysteroscopy, efficacy, safety

## Abstract

**Objective:**

Remimazolam besylate and Ciprofol are newer sedatives used in minor surgeries. Propofol is a classic drug mainly used for short surgeries. This trial was conducted to compare the efficacy and safety of remimazolam besylate, ciprofol, and propofol during hysteroscopic surgeries.

**Methods:**

Patients undergoing hysteroscopy were randomly assigned to receive remimazolam besylate (Group R), ciprofol (Group C), or propofol (Group P). A total of 194 patients were assessed for eligibility. One patient in Group P was excluded because the operation had timed out of 60 min. Patients all in Group R、Group C and Group P received an induction dose of 0.2 mg/kg remimazolam besylate、0.4 mg/kg ciprofol、2.0 mg/kg propofol seperately over 30 s. A corresponding dosage of 1 mg/kg/h、0.6–1.2 mg/kg/h and 3.0–6.0 mg/kg/h was given by continuous intravenous infusion to maintain a BIS of 40–60 till the end of the surgery. The incidence rates of body movement, respiratory depression, and adverse effects were compared among the groups.

**Results:**

The incidence of injection pain was much higher in Group P (64.1%) than that in Group R (3.4%) and Group C (3.2%, both *P* < 0.001). The onset time was significantly shorter in Group P than that in Group R and Group C (both *P* < 0.01). The awakening time (MOAA/S score = 3) was longest in Group R, followed by Groups C and Group P (*P* < 0.01). The time to complete recovery (MOAA/S score = 5) has no significantly difference between Group C and Group P, whereas the onset time was significantly shorter in Group R (*P* < 0.01). The number of body movements was significantly higher in Group R than that in Group C and Group P (*P* < 0.01). The incidence of hypotension was significantly lower in Group R than that in Group C and Group P (both *P* < 0.01). The rate of respiratory inhibition was significantly lower in Group R and Group C than that in Group P (both *P* < 0.05).

**Conclusion:**

Considering jointly the safety and efficacy, ciprofol seems to be the best choice for sedation.

## 1 Introduction

Hysteroscopy is one of the most common outpatient procedures in the diagnosis and treatment of endometrial and other intrauterine diseases. Most patients require anaesthetic intervention because they cannot tolerate the intensive pain of hysteroscopic operation (Zhang F. et al., 2022).

The commonly used anaesthetic regimen for hysteroscopic surgery is short-acting sedatives combined with opioids (Fan et al., 2023). Remimazolam besylate and ciprofol are new short-acting sedatives, whereas propofol is a classic one (Liu et al., 2022; Zhang J. et al., 2022). Propofol, combined with opioids, remains the most commonly method to control pain during hysteroscopy (Tang et al., 2023). However, propofol has a high incidence of adverse events, such as injection pain, hypotension, and respiratory inhibition (Zhong et al., 2022).

Both remimazolam besylate and ciprofol are newer sedatives with a quick onset、a short maintenance and recovery time (Zhang et al., 2021; Li et al., 2022). Remimazolam besylate and ciprofol have been reported as safer alternatives during hysteroscopy (Tang et al., 2022; Lan et al., 2023). However, researches have not yet compared the efficacy and safety of remimazolam besylate, ciprofol, and propofol.

This trial was conducted to confirm the efficacy and safety of remimazolam besylate, ciprofol, and propofol during hysteroscopy.

## 2 Materials and methods

### 2.1 Patients

This prospective, randomized, double-blind, non-inferiority trial was carried out at Lishui people’s Hospital. This study was approved by the Clinical Research Ethics Committee of Lishui people’s Hospital (LLW-FO-403) and registered at http://www.chictr.org.cn (ChiCTR2300069105). The first registration was 07/03/2023. Written informed consent was obtained from patients undergoing elective hysteroscopy at Lishui people’s Hospital from 10/03/2023 to 31/07/2023. We confirmed that all research was performed in accordance with relevant guidelines, and included in their manuscript a statement confirming that informed consent was obtained from all participants. Data is provided within the supplementary information files.

The inclusion criteria were an age of 18–65, American Society of Anesthesiologists (ASA) physical status I or II, and body mass index (BMI) of 19–30 kg/m^2^. Patients were excluded if they met any of the following criteria: (1) planned endotracheal intubation or laryngeal mask placement; (2) acute heart failure, unstable angina pectoris, myocardial infarction occurred within 6 months prior to screening, resting electrocardiograph (ECG) heart rate (HR) < 50 beats/min, grade III atrioventricular block, severe arrhythmia, moderate-to-severe heart valve disease, QTc of ≥450 ms for men and ≥470 ms for women; (3) severe respiratory disease (e.g., obstructive sleep apnoea syndrome, acute respiratory infection, acute onset of chronic obstructive pulmonary disease, uncontrolled asthma); (4) psychiatric disorders (e.g., schizophrenia, mania, bipolar disorder, mental disorder), a long history of psychotropic drug use, or cognitive dysfunction; (5) difficulty regarding respiratory management (grade IV Modified Mallampati Score); (6) anaemia or thrombocytopenia [haemoglobin <90 g/L, platelet <80 × 10^9^/L]; (7) liver dysfunction [aspartate aminotransferase and/or alanine aminotransferase ≥2.5 × upper limit of normal (ULN), total bilirubin ≥1.5 ULN] or renal dysfunction (urea or urea nitrogen ≥1.5 × ULN, serum creatinine > ULN); (8) history of drug abuse or alcohol abuse within 2 years before screening [alcohol abuse: average daily consumption of more than two single units (1 unit = 360 mL of beer, 45 mL of 40% liquor, or 150 mL of wine); (9) uncontrolled blood pressure [in screening stage, sitting systolic blood pressure (SBP) ≥ 160 mmHg or ≤90 mmHg and/or diastolic blood pressure (DBP) ≥ 100 mmHg]; (10) use of benzodiazepines and/or opioids for nearly 3 months; (11) allergy or contraindication to benzodiazepines, opioids, propofol, and their components; (12) pregnancy or breastfeeding in women or planning to be pregnant within 3 months (including men); (13) participation in any clinical trial within the last 3 months; and (14) the surgery lasting more than 60 min.

### 2.2 Randomization and blinding

**FIGURE 1:**
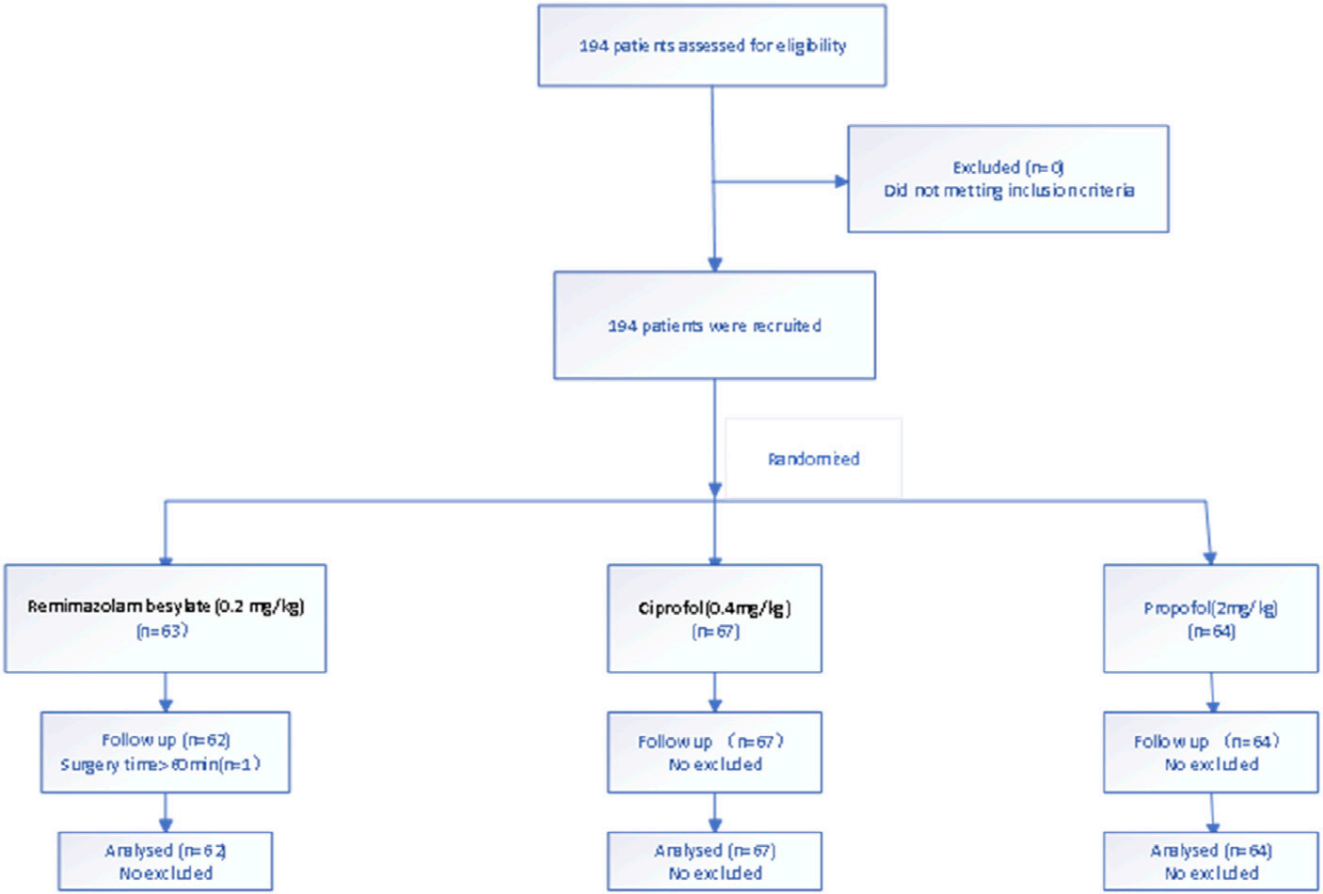
Flow diagram representing patient enrollment, group assignment, and analysis.

Patients were randomly assigned into the remimazolam (Group R), ciprofol (Group C), or propofol (Group P) by computer-generated randomization. A total of 194 patients were assessed for eligibility. One patient in the propofol group was excluded because the operation time exceeded 60 min. Therefore, data for 62 patients in Group R, 67 patients in Group C and 64 patients in Group P were analyzed. Te study fowchart is shown in Figure 1.

### 2.3 Anesthesia

All patients received no premedication before being transferred to the operating room (OR). ECG, non-invasive blood pressure including systolic blood pressure (SBP), diastolic blood pressure (DBP), mean blood pressure, SpO2, and HR were monitored upon arrival to the OR. Prophylactic oxygen (5 L/min) was provided by a suitable face mask during the operation. All patients received sufentanil citrate (Yichang Humanwell Pharmaceutical Co., Ltd., Yichang, China) at 0.1 μg/kg intravenously for analgesia before the start of hysteroscopy. Patients were also monitored by a bispectral index (BIS) sensor (ConView YY-106, Pearlcare, Zhejiang, China) positioned on the forehead. BIS of between 40 and 60 reflects adequate hypnotic effects of general anaesthesia with reasonably rapid recovery of consciousness.

Anaesthesia was administered by a board-certified anaesthesiologist, and all procedures were performed by the same group of experienced gynaecologists.

The following evaluation time points were used:

T0: Pre-anaesthesia.
T1: Two minutes after initiation of the sedative infusion.
T2: Cervical dilatation.
T3: End of the operation.
T4: Initial awakening time (MOAA/S score = 3).
T5: Complete recovery (MOAA/S score = 5).

After surgery, the patients were transferred to the post-anaesthesia care unit for close monitoring until discharge. Standard monitoring comprised mean arterial pressure (MAP), HR, and SpO2. Patients were discharged to the general ward when their Aldrete score reached at least 9.

All patients in Group R received an induction dose of 0.2 mg/kg remimazolam besylate (Yichang Humanwell Pharmaceutical) over 30 s and a maintenance dosage of 1 mg/kg/h by continuous intravenous infusion was also conducted to maintain a BIS of 40–60 until the end of surgery. All patients in Group C received an induction dose of 0.4 mg/kg ciprofol (Haisco Pharmaceutical Group Co., Ltd., Liaoning, China) over 30 s and a maintenance dosage of 0.6–1.2 mg/kg/h by continuous intravenous infusion. A BIS between 40 and 60 was aimed to be maintained until the end of surgery. All patients in Group P received an induction dose of 2.0 mg/kg propofol (Fresenius Kabi AG, Graz, Austria) over 30 s. The propofol infusion rate was set at 3.0–6.0 mg/kg/h to maintain the sedation. When BIS <60 and the eyelash reflex was completely lost, the surgery started.

In the case of body movement that affected the operation, propofol 0.5 mg/kg/time was added in Group P, ciprofol 0.1 mg/kg/time was added in Group C, and remimazolam besylate 0.05 mg/kg/time was added in Group R. If the depth of sedation was insufficient (BIS >60), the patients then received an intravenous injection of propofol 0.5 mg/kg/time in Group P, ciprofol 0.1 mg/kg/time in Group C, and remimazolam besylate 0.05 mg/kg/time in Group R until the BIS was maintained at 40–60.

When adverse hemodynamic events, including hypotension (SBP <90 mmHg, DBP <50 mmHg, or MAP decrease of ≥20% from baseline) and bradycardia (HR < 50 beats/min or a decrease in HR of ≥20% from baseline) occurred during the procedure, ephedrine or atropine was administered for treatment. If respiratory depression occurred, as indicated by SpO2 <90% or respiratory rate <8 breaths/min for more than 1 min, or airway obstruction or apnoea was detected, mandibular elevation or mechanical ventilation was performed.

### 2.4 Outcomes

The primary outcome of this study was the incidence of various adverse events, such as injection pain, low SpO2, bradycardia, and hypotension (defined in Table 2). These events were treated by intravenous ephedrine or atropine or mask ventilation.

The secondary outcomes included the rate of body movements, the number of body movements, the change in haemodynamics, recovery time (MOAA/S score = 3 and 5), anaesthetist’s satisfaction, and time to disappearance of the eyelash reflex. The rate of body movements only differed between Groups P and R, being lower in the former group (*P* < 0.05). The number of body movements was higher in Group R than in Groups P and C (both *P* < 0.01). The number of body movements did not differ between Groups P and C (Table 2). We searched relevant literature and found that MOAA/S scores were used as the evaluation method of anaesthetic depth in most studies.

### 2.5 Statistical analysis

In the pilot study on the combined use of propofol and sufentanil in hysteroscopy, the incidence of various intraoperative adverse events was 30%. This result of our small pre-experiment indicated a clinically significant reduction in the incidence of adverse events to 5% with the use of sufentanil and to 8% with the use of ciprofol. The calculated sample size for each group was 62 participants, and the significance level was *P* < 0.05 (a = 0.05). Given a 10% attrition rate, the strength was 80% (b = 0.20) (Teng et al., 2021; Rex et al., 2021).

SPSS version 20.0 (IBM Corporation, Armonk, NY, United States) statistical software was used to analyse the data. Normally distributed data were presented as the mean and standard deviation. One-way ANOVA was used to compare the measurements among the groups. Categorical variables were expressed as frequencies (percentage) and analyzed using Pearson’s chi-squared test.

## 3 Results

### 3.1 Patient demographic characteristics

The demographic and surgical characteristics of the patients are presented in Table 1. The characteristics of the patients, including age, ASA physical status, and BMI, were similar among the groups. There were also no significant differences in the duration of the procedure, MAP, and HR among the groups before surgery.

**TABLE 1:** Demographic and clinical data for each group.

	Group R (n = 62)	Group C (n = 67)	Group P (n = 64)
Age (years)	37.0 ± 9.9	37.5 ± 9.5	36.0 ± 8.7
BMI (kg/m2)	23.0 ± 2.6	22.7 ± 2.8	22.2 ± 2.9
ASA (I/II) (n)	32/30	35/32	31/33
Duration of operation (sec)	1,295 ± 516	1,202 ± 458	1,342 ± 505
MAP (mmHg)	82.9 ± 9.1	82.7 ± 8.8	83.0 ± 8.3
HR (bpm)	73.7 ± 11.7	72.1 ± 10.2	70.9 ± 9.8

### 3.2 Adverse events

The number of adverse events was 8 (3.2%) in Group R, 22 (8.2%) in Group C, and 65 (25.4%) in Group P (*P* < 0.01, Table 2). During the surgical process, no patient in Group R reported injection pain. In Group C, one patient experienced injection pain. In Group P, 29 (45.3%) patients experienced injection pain, which was higher than the numbers of patients in Groups R and C (both *P* < 0.01). Meanwhile, 4 (6.5%) 5 (7.5%), and 13 (20.3%) patients in Groups R, C, and P, respectively, experienced respiratory suppression. The rates of respiratory suppression were significantly lower in Groups P and C than in Group R (both *P* < 0.05). Meanwhile, 3 (4.8%) patients in Group R, 14 (20.9%) patients in Group C, and 21 (32.8%) patients in Group P experienced hypotension. The rate of hypotension was significantly lower in Group R than in Groups C and P (both *P* < 0.01). The rate did not differ between Groups C and P.

**TABLE 2:** The definitions and incidence of adverse events.

Adverse events	Definitions	Group R (n = 62)	Group C (n = 67)	Group P (n = 64)
Injection pain	“Subjective” assessment, patients verbally reported their pain by themselves after the first injection	0 (0%)**	1 (1.5%)**	29 (45.3%)
Low SpO2	Intraoperative SpO2 ≤ 90%	4 (6.5%)*	5 (7.5%)*	13 (20.3%)
Bradycardia	Intraoperative HR < 50 bpm	1 (1.6%)	2 (3.0%)	2 (3.1%)
Hypotension	SBP <90 mmHg, DBP <50 mmHg, or mean arterial pressure (MAP) decrease of ≥20% below baseline	3 (4.8%)**^★^	14 (20.9%)	21 (32.8%)
Total incidence of adverse events		8 (3.2%)**^▲^	22 (8.2%)**	65 (25.4%)
Body movement	Visible hand bending or head movement	44 (71.0%)*	43 (64.2%)	33 (51.6%)
The number of body movement		1.75 ± 1.64**^★^	0.99 ± 0.99	0.86 ± 1.11

*P < 0.05, **P < 0.01 compared with Group P

^★^ *P* < 0.01, ^▲^ *P* < 0.05 compared with Group C.

### 3.3 Sedation-related outcomes

All three groups of patients were successfully sedated. The time of disappearance of the eyelash reflex was significantly shorter in Group P than in Groups R and C (both *P* < 0.01), whereas this variable did not differ between Groups R and C. The initial awakening time (MOAA/S score = 3), was significantly shorter in Group P than in Group R and C (both *P* < 0.01), whereas the time was shorter in Group C than in Group R (*P* < 0.05). The full awakening time (MOAA/S score = 5) was significantly shorter in Group R than in Group C and P (both *P* < 0.01), but no difference was detected between Groups C and P (Table 3).

**TABLE 3:** Sedation-related outcomes.

	Group R (n = 62)	Group C (n = 67)	Group P (n = 64)
The success rate of sedation (%)	100.0	100.0	100.0
The time to loss eyelash reflex (sec)	34.4 ± 16.5**	35.4 ± 17.3**	25.5 ± 15.4
Initial awakening time (MOAA/S score = 3) (sec)	395.9 ± 280.4**^▲^	310.0 ± 125.6**	256.6 ± 95.8
Full awakening time (MOAA/S score = 5) (sec)	813.0 ± 358.8**^★^	670.1 ± 218.5	643.4 ± 209.9

**P < 0.01 compared with Group P

^★^ *P* < 0.01, ^▲^ *P* < 0.05 compared with Group C.

### 3.4 Changes in circulation

**FIGURE 2:**
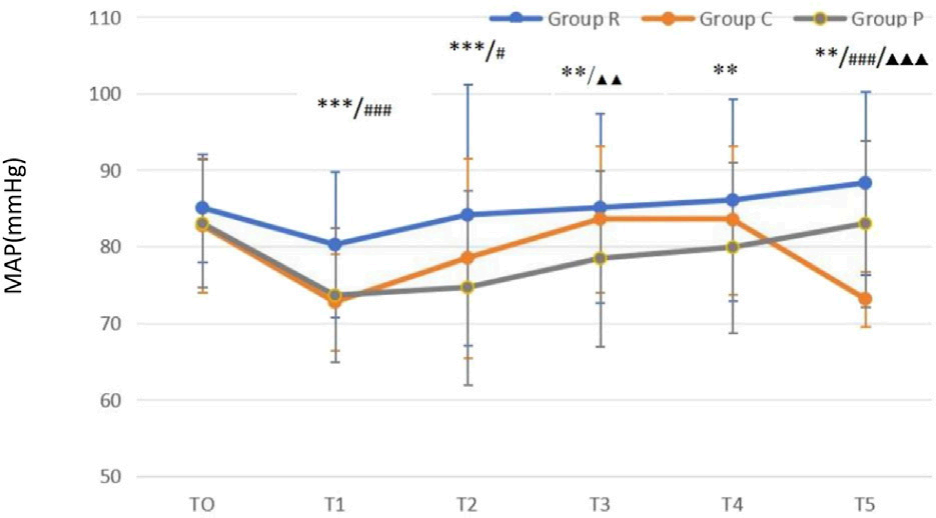
Mean arterial pressure (MAP)-time graph **P < 0.01 Group R vs. Group P; ***P < 0.001 Group R vs. Group P; #*P* < 0.05 Group C vs. Group R; ###*P* < 0.001 Group C vs. Group R; ▲▲*P* < 0.01 Group C vs. Group P; ▲▲▲*P* < 0.001 Group C vs. Group P.

**FIGURE 3:**
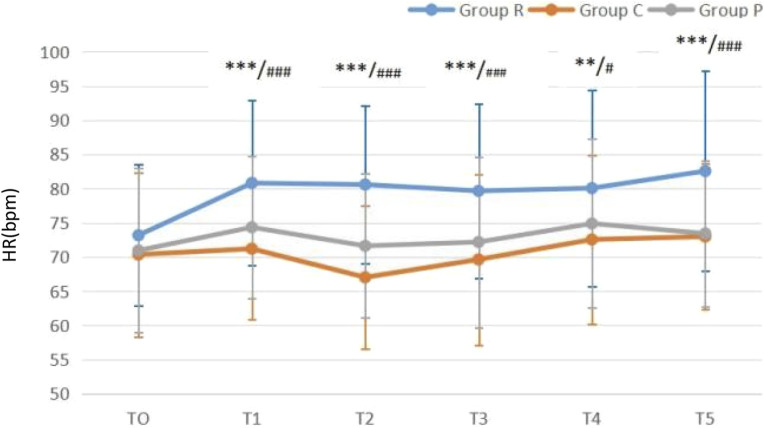
Heart rate (HR)-time graph **P < 0.01 Group C vs. Group R; ***P < 0.001 Group C vs. Group R; #*P* < 0.05 Group P vs. Group R; ###*P* < 0.001 Group P vs. Group R.

Compared with that at T0, MAP was all reduced in all three groups (all *P* < 0.05) after anesthetics induction, but all values were within the clinically normal range. Excluding the unexpected decrease in MAP when patients were completely awake, MAP gradually increased over times in all groups (Figure 2). Compared with that at T0, HR was all increased in all three groups (all *P* < 0.05) after anesthetics induction. After anesthetics induction, HR remained higher in Group R than in Groups C and P, whereas no difference was noted between Groups C and P (Figure 3).

## 4 Discussion

This trial demonstrated that remimazolam besylate, ciprofol, and propofol could be successfully used for sedation in hysteroscopic surgery. All three drugs have their own advantages and disadvantages. Based on our data, remimazolam besylate has the highest safety, followed by ciprofol and propofol. In terms of sedative efficacy, propofol appeared best, followed by ciprofol and remimazolam besylate.

Propofol has been the most commonly used intravenous anaesthetic drug for the induction and maintenance of anaesthesia and sedation of patients because of its rapid onset, clearance, and patient recovery (van der Meulen et al., 2023). However, there are some unavoidable limitations to its usage, such as injection pain, suppression of circulatory function, and respiratory depression (Kim and Fechner, 2022). Previous studies illustrated that remimazolam besylate was characterized by a pharmacokinetic–pharmacodynamic profile with a rapid onset, rapid recovery, and moderate hemodynamic side effects (Deng et al., 2022). Ciprofol is a novel 2,6-disubstituted phenol derivative developed for the induction and maintenance of anaesthesia, and it exhibited an improved anaesthetic profile and less injection pain than propofol in pre-clinical studies. Ciprofol is also a gamma-aminobutyric acid type A receptor agonist (Chen et al., 2022).

Remimazolam besylate and ciprofol cause little injection pain, whereas the incidence of injection pain was much higher for propofol (45.3%). The mechanism of propofol-induced injection pain is currently unclear. The presence of free propofol in blood could be the main factor causing injection pain. High concentrations of free propofol directly stimulate the intima of the venous wall and transmit pain signals to the central nervous system through Aδ fibres, causing pain at the injection site (Tan et al., 2022). The reported incidence of propofol injection pain varies widely, ranging from 30% to 70% (Gan et al., 2024), in line with our findings.

This study found that the incidence of respiratory inhibition was much higher for propofol (20.3%) than for remimazolam besylate (6.5%) and ciprofol (7.5%). This is related to the inhibitory effect of propofol on the respiratory centre (Guo et al., 2022). Because respiratory depression is a critical issue during anaesthesia, the lower incidence of respiratory depression associated with remimazolam besylate and ciprofol makes them potentially safer options than propofol for hysteroscopic procedures.

Intraoperative hypotension is a common complication of hysteroscopic surgery (Zhang et al., 2021). In a previous multicentre phase III clinical trial in China, 384 eligible patients undergoing colonoscopy were randomly assigned to receive remimazolam or propofol. It has been reported that the incidence of hypotension is lower in patients treated with ciprofol than in those treated with propofol. The decrease in blood pressure was also significantly smaller in the ciprofol group (Lan et al., 2023). Our study found that remimazolam had a lower incidence of hypotension (23.71%) than propofol (51.05%) (Fang et al., 2023). Our study additionally revealed that propofol (32.8%) and ciprofol (20.9%) endure a higher risk of hypotension than remimazolam besylate (4.8%) during surgery. Although the incidence of hypotension did not significantly differ between propofol and ciprofol, the incidence was numerically lower for ciprofol. This could be related to the weaker sedative effect of remimazolam besylate.

There was obvious limb bending or head movements in all three groups during surgery, with these incidents occurring more frequently in Group R than in Group P. The probability of these body movement did not significantly differ between Groups P and C or between Groups C and R. The number of intraoperative body movements was significantly higher in Group R than that in Groups P and C, whereas no difference was detected between Groups P and C. Inadequate intraoperative analgesia and pain caused by surgical stimulations might contribute to these involuntary movement. Although this study used a single intravenous injection of sufentanil for intraoperative analgesia, its effect decreased over time, and no additional doses were administered during the operation. In previous studies, continuous intravenous infusion of remifentanil was used for pain control (Zhang et al., 2021).

Our study found that the time to loss of the eyelash reflex was shorter in Group P than in Groups R and C. There was no significant difference in this variable between Groups R and Group C. This indicates that propofol has a shorter onset than remimazolam besylate and ciprofol. This is consistent with previous reports which suggested that propofol has a shorter onset time than remimazolam besylate (Liu et al., 2021). It has been reported that the time to loss of the eyelash reflex is significantly longer for ciprofol than for propofol (Lan et al., 2023). Up to now, no study has compared the time to loss of the eyelash reflex between remimazolam besylate and ciprofol.

The initial awakening time was fastest for propofol, followed by ciprofol and remimazolam besylate. However, the full awakening was slowest for remimazolam besylate, with no difference between propofol and ciprofol. However, prior research found no significant difference in the awakening time between propofol and remimazolam (Zhang S. et al., 2022). This could be attributable to the use of different scoring standards—we used MOAA/S scoring and the prior study used MMSE scoring.

In conclusion, remimazolam besylate had the best safety profile, followed by ciprofol and propofol. In terms of sedative efficacy, propofol appeared to be best, followed by ciprofol and remimazolam besylate. In regards to both safety and efficacy, ciprofol appears to be the optimal drug. Because of its strong efficacy and similar respiratory side effects as remimazolam besylate, ciprofol has a higher circulating side effect rate than remimazolam, which is within an acceptable range at the same time.

## Data Availability

The datasets presented in this study can be found in online repositories. The names of the repository/repositories and accession number(s) can be found in the article/supplementary material.
